# Epidemiological trends in nosocomial candidemia in intensive care

**DOI:** 10.1186/1471-2334-6-21

**Published:** 2006-02-10

**Authors:** Matteo Bassetti, Elda Righi, Alessandro Costa, Roberta Fasce, Maria Pia Molinari, Raffaella Rosso, Franco Bobbio Pallavicini, Claudio Viscoli

**Affiliations:** 1 Infectious Diseases Department, S. Martino Hospital and University of Genoa, Genoa, Italy; 2 Intensive Care Unit, S. Martino Hospital, Genoa, Italy

## Abstract

**Background:**

Infection represents a frequent complication among patients in Intensive Care Units (ICUs) and mortality is high. In particular, the incidence of fungal infections, especially due to *Candida spp.*, has been increasing during the last years.

**Methods:**

In a retrospective study we studied the etiology of candidemia in critically ill patients over a five-year period (1999–2003) in the ICU of the San Martino University Hospital in Genoa, Italy.

**Results:**

In total, 182 episodes of candidaemia were identified, with an average incidence of 2.22 episodes/10 000 patient-days/year (range 1.25–3.06 episodes). Incidence of candidemia increased during the study period from 1.25 in 1999 to 3.06/10 000 patient-days/year in 2003. Overall, 40% of the fungemia episodes (74/182) were due to *C.albicans*, followed by *C. parapsilosis*(23%), *C.glabrata *(15%), *C.tropicalis *(9%) and other species (13%). Candidemia due to non-*albicans *species increased and this was apparently correlated with an increasing use of azoles for prophylaxis or empirical treatment.

**Conclusion:**

The study demonstrates a shift in the species of Candida causing fungemia in a medical and surgical ICU population during a 5 year period. The knowledge of the local epidemiological trends in *Candida *species isolated in blood cultures is important to guide therapeutic choices.

## Background

*Candida *is an increasing cause of bloodstream infection (BSI), causing significant mortality and morbidity, especially in non-neutropenic critically ill patients. Its overall incidence raised fivefold in the past ten years and *Candida spp*. is currently between the fourth and the sixth most common nosocomial bloodstream isolate in American and European studies [1,2]. Despite the availability of effective antifungal therapy, mortality in the last decade remained high, ranging from 36% to 63% [3]. In terms of species of *Candida*, recently, a shift towards *non-albicans *species was reported by some authors especially in hematological and transplanted patients [4-7]. Some of these emerging species has been correlated with increased virulence [8], and sometimes, but not always, with increased mortality [9]. An increasing role for non*-albicans *species was also noticed in studies performed among ICU patients, although the issue is somewhat controversial [3,10-12].

As shown by the SENTRY antimicrobial surveillance program [1], *C. albicans *is still the species most frequently isolated in BSI [13,14], while in other groups of patients *non-albicans *species have surpassed *C.albicans *as a cause of candidemia. *C.parapsilosis *and *C.tropicalis *are isolated more frequently than *C.albicans *in some European and Latin American centres [15].

A reduced antifungal susceptibility in *non-albicans *species and a correlation with routine fluconazole prophylactic use has been suggested [5,16]. Intrinsic and emerging resistance of *non-albicans *species to azoles actually represents a major challenge for empirical therapeutic and prophylactic strategies [17].

We have performed a retrospective study of *Candida spp*. BSI in the ICU of the San Martino University Hospital, in Genova, Italy, between 1999 and 2003, with the double aim of understanding if any change was detectable in the distribution of the various *Candida *species as cause of BSI over the years and of estimating whether this change, if existing, was correlated with the use of fluconazole for prophylaxis or therapy.

## Methods

The ICU of the San Martino General Hospital in Genova, Italy, is a mid-size medical and surgical unit with 18 beds and about 500 admissions per year. Patients who developed a clinically and microbiologically documented candidemia were identified through a microbiological laboratory survey and data were recorded in an electronic data base. The patients chart review was performed in order to identify clinically relevant episodes. Candidemia was defined as at least one positive blood culture yielding Candida spp. in a patients with fever or other clinical signs of infection. Nosocomial candidemia was defined as a candidemia occurring at least 48 h after admission. During the study period there were no changes in microbiological laboratory techniques. Candida species were isolated from blood using BACTEC 860 system (Becton Dickinson, INC, Sparks, MD). The species were identified using API-32C system (bioMerieux Vitek, Inc, St. Louis, MI). Frequencies and descriptives of demographic and clinical characteristics of the patient population were determined.

All patients were evaluable for the inclusion in this study. The Chi-square-test or the Fisher Exact-test were used to compare categorical variables. Chi-square-test for trend was used to estimate the relationship between *albicans *and non-*albicans *isolation rates during the years of study. In order to understand whether or not there was a correlation between the distribution of the different *Candida *species causing BSI and the use of fluconazole, the DDD of fluconazole per 1000 patient-days was calculated and plotted against the type of isolated *Candida *on a yearly basis. A logistic regression analysis between *C. albicans *rate and fluconazole usage was then performed.*P *values < 0.05 were considered significant.

All the patients consented to participation in the study and publication of the results.

## Results

**Figure 1:**
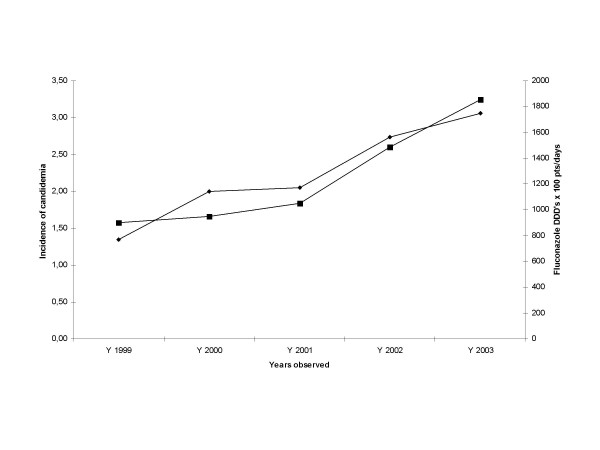
◊ incidence of candidemia episodes/10 000 patient-days/year; ■ DDD's of fluconazole × 100 pts/days.

**Figure 2:**
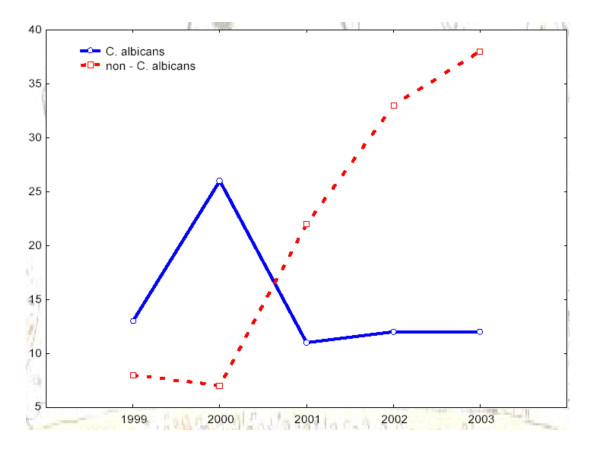
L*ine plot *representation of *Candida albicans *and *Candida non-albicans *isolates rates during the study period.

In total, 182 episodes of candidaemia were identified, with an average incidence of 2.22 episodes/10 000 patient-days/year (range 1.25–3.06 episodes), as shown in table 1. Incidence of candidemia increased during the study period from 1.25 in 1999 to 3.06 episodes/10 000 patient-days/year in 2003 (Figure 1). The demographic and clinical characteristics of the patients are summarized in table 1. Patients with *Candida *non-albicans fungemia were significantly older. There were no significant differences in sex, age, central venous, catheterization, parenteral nutrition, ventilator dependence, Apache II score and other underline disease. Non-albicans candidemia are significantly more frequent in patients with solid tumor. Overall, 40% of the episodes (74/182) were due to *C.albicans*, followed by *C. parapsilosis*(23%), *C.glabrata *(15%), *C.tropicalis *(9%) and other species (13%) (Table 2) The percentage of *C. albicans *which was isolated in about 60% of the episodes in the first years of study, progressively dropped to 24%, with a median percentage year reduction of 13%, while the absolute number and the incidence of *C.albicans *did not vary during the years observed. The change was statistically significant (K^2 ^= 24.452; p < 0.0001), although in absence of a rigorous linear trend. Non*-albicans *strains steadily increased throughout the study period surpassing *C.albicans *in the second half of the study and raising from 21% to 67% (P = 0.01) (Figure 2). The proportion of non*-albicans *species went from 38% and 21% of total isolates in the years 1999–2000 and then to 67%, 73% and 76% in 2001, 2002 and 2003, respectively. Among non*-albicans *strains, in 2002 we noted an increase of *C.parapsilosis *isolation rates, which reached 51% of total, while absence of *C.tropicalis *was noted in the years 2000–2001.

**Table 1:** Patient characteristics of 182 patients with candidemia.

**Characteristics**	*C.albicans *(n= 74)	*C. non albicans *(n= 108)	P value
Male	41	70	NS
Famale	33	38	NS
Age (years) mean ± SD Range	58.4 ± 15.4	64.4 ± 11.4	<0.05
Central venous catheterization	65/74	99/108	NS
Parenteral nutrition	38/74	64/108	NS
Ventilator dependence mean ± SD Range	27.9 ± 23.6	32.6 ± 24.5	NS
Apache II score mean ± SD Range	21.8 ± 7.4	25.6 ± 9.8	NS
**Underline disease**			
Surgery	33/74	40/108	NS
Solid tumor	11/74	35/108	<0.05
Solid organ transplant	8/74	10/108	NS
Trauma	7/74	9/108	NS
Hematological malignancies	6/74	10/108	NS
Burn	4/74	3/108	NS

Others	5/74	1/108	NS

NS = Not significant

**Table 2:** Incidence and distribution of candidemia in the years 1999 – 2003 in S. Martino Hospital ICU expressed in number and percentage of isolates. Polymicrobial infections were excluded.

	**1999**	**2000**	**2001**	**2002**	**2003**	**Total**
	**Number of isolates (percentage of species in the year)**					

***C.albicans***	13 (62)	26 (79)	11 (33)	12 (27)	12 (24)	74 (40)
***C.parapsilosis***	2 (9)	0	4 (12)	23 (51)	13 (26)	42 (23)
***C. glabrata***	0	1 (3)	12 (36)	2 (4)	12 (24)	27 (15)
***C. tropicalis***	5 (24)	0	0	7 (16)	5 (10)	17 (9)
**Othres (*C.kruzei, C.guillermondii, C.lusitanae*)**	1 (5)	6 (18)	6 (19)	1 (2)	8 (16)	24 (13)
**Total candidemia**	21	33	33	45	50	182
**Incidence of candidemia/10 000 patient-days/year**	1.25	2	2.05	2.74	3.06	2.22
**Incidence of candidemia due to *C.*albicans/10 000 patient-days/year**	0.84	1.58	0.66	0.73	0.73	0.91

**Figure 3:**
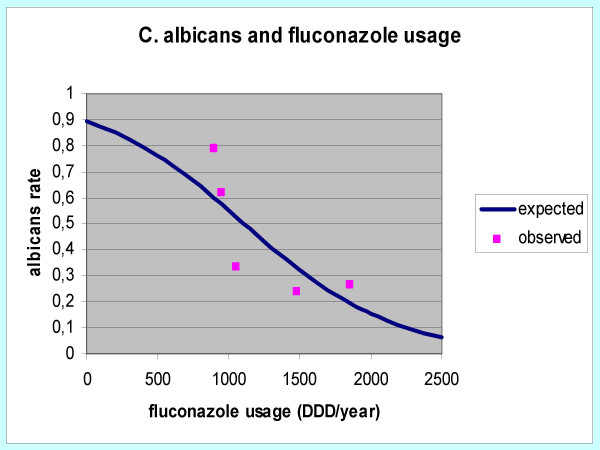
Correlation using logistic regression between percentage reduction of *C. albicans *isolation rates and fluconazole use [DDDs per year].

The use of fluconazole for prophylaxis or therapy increased four times during the study period. Logistic regression analysis showed a statistically significant correlation between the shift from *albicans *to non-*albicans *strains and the yearly fluconazole consumption. As shown in figure 3, an increase of 100 fluconazole DDDs corresponded to a reduction in the isolation of *C. albicans *of 5%.

## Discussion

Our study demonstrates a shift in the species of Candida causing fungemia in a medical and surgical ICU population during a 5 year period. The proportion due to C. *albicans *decreased whereas that due to other species, such as *C. parapsilosis*, *C. tropicalis *and *C.glabrata*, increased. Such an increase in non-albicans species was also seen in retrospective reviews of candidemias [4,10,12,15,16]. In contrast to hematologic patients where a decrease in *C.albicans *infection resulted in a significant reduction in the incidence of candidemia [17], in our experience the overall incidence actually increased (Table 1; Figure 1). Traditionally, *C.tropicalis *has been the second and *C.glabrata *the third or fourth most common Candida species recovered from blood [4,8]. In our study *C.parapsilosis *surpassed the other non-albicans to become the most common species isolated after *C.albicans*. The high incidence of *C.parapsilosis *candidemia has been previously reported in South American hospitals [15,18,19]. The role of *C.parapsilosis *as an exogenous acquired pathogen is well known and has been associated with parenteral alimentation and intravascular devices [20], commonly used in critical patients.

Several investigators postulated that the widespread use of fluconazole would have selected yeast species intrinsically resistant or less sensitive to fluconazole, such as *Candida kruzei, C. glabrata or C. tropicalis *[21-23]. Some published reports confirmed this hypothesis, while others did not [2,24,25]. At the San Martino Hospital, the incidence of infections caused by most non-albicans Candida species changed substantially during the study period. These changes occurred in concomitance with a four fold increase in the use of fluconazole. In our ICU, during 2001 and the following years, the usage of fluconazole at dosage of 200–400 mg/day increased because of the changing in prophylactic strategy in high-risk patients, according to the efficacy demonstrated in various reports [26,27]. A logistic regression analysis showed a statistically significant correlation between the shift from *albicans *to non-*albicans *strains and the yearly fluconazole consumption, as shown in figure 2. However, it be recognised that other events might have played a role in the selection of different species. For example, in the last 2 years of the study, the increased proportion of candidemias due to *C.parapsilosis*, a yeast species almost always susceptible to fluconazole, is not readily explained by increased fluconazole use. It is likely that changes in the proportion of fungemias due to *C. parapsilosis *reflect nosocomial acquisition of this species.

In ICU, the role for the use of fluconazole remains controversial, since fluconazole was demonstrated to reduce the incidence of *Candida *infections in a particular group of high-risk patient population [26-28].

Though limited by its retrospective nature, our study focuses the attention on predominance of *non-C.albicans *isolates, usually less susceptible or intrinsically resistant to fluconazole.

It is well known that positive blood culture for *Candida spp*. is a life threatening situation, requiring an empirical antifungal treatment which should started with the appropriate agents as soon as possible. Therefore the knowledge of the local epidemiological trends in *Candida *species isolated in blood cultures is important to guide therapeutic choices.

The data we have reported are based on records from the microbiology laboratory, and thus, have certain inherent limitations. Our study did not address specific risk factors, which undoubtedly play a role in the selection of species causing fungemia. However, this type of study does reveal overall long-term trends that should be helpful to physicians and antibiotic use committees in establishing guidelines for the appropriate use of antifungal agents.

## Competing interests

The author(s) declare that they have no competing interests.

## Authors' contributions

MB: Data acquisition, data/statistical analyses, drafting the manuscript

ER: Sample collection, drafting the manuscript, critically revising for medical content

AC: Sample collection, critically revising for medical content

RF: Sample collection, critically revising for medical content

MPM: Data acquisition, data analyses

RR: Study design and conception, critically revising for medical content

FBP: Study design, conception and coordination

CV: Study design, conception and coordination

All authors contributed to writing of the final manuscript.

All authors read and approved the final manuscript

## Pre-publication history

The pre-publication history for this paper can be accessed here:


